# miR-34a targets PAI-1 to regulate urinary microalbumin and renal function in hypertensive mice

**DOI:** 10.1186/s40001-020-00404-7

**Published:** 2020-03-17

**Authors:** Ruitao Liu, Lihong Yang, Qingmin Wei

**Affiliations:** Department of Cardiovascular Medicine, Xingtai People’s Hospital, No.16 Hongxing East Street, Qiaodong District, Xingtai, 054000 Hebei China

**Keywords:** miR-34a, PAI-1, Hypertension, Urinary microalbumin, Renal function

## Abstract

**Background:**

The aim of the study is to investigate the effects of miR-34a targeted at PAI-1 on urinary microalbumin and renal function in hypertensive mice.

**Methods:**

Twenty specific-pathogen-free (SPF) BPN/3J mice were selected in normal group, and 120 SPF BPH/2J mice were evenly divided into model group, negative control group, miR-34a mimic group, miR-34a inhibitor group, Si-PAI-1 group, and miR-34a inhibitor + Si-PAI-1 group. qRT-PCR was used to detect the expression of miR-34a and PAI-1 mRNA. The protein expressions of PAI-1, angiotensin-converting enzyme (ACE) and ACE2 were detected by Western blot. Serum levels of AngII and Ang1-7 were detected by ELISA.

**Results:**

miR-34a negatively regulated the expression of PAI-1. Compared with the normal group, mice in the other groups had significantly lower body weight, increased systolic blood pressure and 24-h urinary microalbumin content, decreased miR-34a expression, superoxide dismutase (SOD) and nitric oxide (NO) content, and ACE2 protein expression, and increased PAI-1 expression, serum creatinine (Scr), blood urea nitrogen (BUN) malondialdehyde (MDA), AngII and Ang1-7 levels, and ACE protein expression (all *P* < 0.05). Compared with the model group, mice in the miR-34a mimic group and Si-PAI-1 group had no significant changes in body weight (all *P* > 0.05), while they had significantly lower systolic blood pressure and 24-h urinary microalbumin content, increased SOD and NO levels and ACE2 protein expression, and decreased PAI-1 expression, Scr, BUN, MDA, AngII and Ang1-7 levels, and ACE protein expression (all *P* < 0.05). Compared with the miR-34a inhibitor group, symptoms in miR-34a inhibitor + Si-PAI-1 group were significantly improved (all *P* < 0.05).

**Conclusions:**

miR-34a can inhibit the expression of PAI-1, thereby reducing urinary microalbumin content in hypertensive mice and protecting their renal function.

## Background

Hypertension, with a high incidence, leads to renal function impairment. The lesions might cause renal failure which is life-threatening [1, 2]. It has been confirmed that the urinary microalbumin content in patients with hypertension is abnormally elevated, which has been regarded as one of the important indicators of hypertension-induced renal damage [3, 4]. At present, only drugs can control hypertension and reduce the risk of complications [5, 6]. What’s more, incomplete understanding of the pathogenesis of hypertension still exists. Exploring and improving the pathogenesis of hypertension are crucial for the treatment of hypertension.

The renal expression of plasminogen activator inhibitor (PAI-1) is abnormally elevated in hypertension-induced renal damage. The overexpression of PAI-1 can result in the accumulation of extracellular matrix by inhibiting the degradation of extracellular matrix, thereby leading to glomerular sclerosis [7–10]. Therefore, finding a negative regulatory factor of PAI-1 is crucial for improving glomerular sclerosis. MicroRNAs, as non-coding RNAs, are widely found in mammals and regulate many life activities [11, 12]. miRNAs regulate life activities indirectly through post-transcriptional regulation. We found targeted binding sites for miR-34a and PAI-1 via the bioinformatics website. It was found that miR-34a is down-regulated in pulmonary hypertension [13–15]. Silencing of miR-34a leads to further development of pulmonary hypertension.

Therefore, we propose that miR-34a acts as a regulator of PAI-1, and then modulates urinary microalbumin and renal function in hypertensive mice. This study is performed to verify this hypothesis.

## Methods

### Animals

A total of 20 BPN/3J mice and 120 BPH/2J mice were chosen, and all mice are 15-week old and specific-pathogen-free [16]. BPH/2 is a hypertensive strain with increased systolic BP in 5th week of life and BPN/3J is control strain to BPH/2. All animals had free access to food and water and were raised in the breeding room with 12 h light. After 1 week, experiments were carried out. This study was approved by the Animal Care and Use Committee of Xingtai People’s Hospital.

### Animal grouping

A total of 20 specific-pathogen-free BPN/3J mice was set as normal group, and 120 BPH/2J mice were evenly divided into model group (without any treatment), negative control (NC) group (injection of NC vector), miR-34a mimic group (injection of miR-34a overexpression vector), miR-34a inhibitor group (injection of miR-34a inhibitor), Si-PAI-1 group (injection of PAI-1 silencing vector), and miR-34a inhibitor + Si-PAI-1 group (combination treatment). All vectors were injected via tail vein at a dose of 2 × 10^8^ U/mL and 200 μL/mouse, 3 times daily for 1 week. 24 h after the finish of the injection, the content of urinary microalbumin in each group was measured. Non-invasive blood pressure method was used to detect systolic blood pressure of mice [17–19]. After the measurement, mice were killed by cutting the tail. A whole blood sample was taken, and the supernatant was collected by centrifugation. The serum was preserved, and the kidney was isolated. The kidneys of 6 mice in each group were randomly immersed in neutral formaldehyde solution for HE staining experiments, and the remaining were stored at − 80 °C for subsequent experiments.

### Dual-luciferase reporter assay

The bioinformatics website (http://www.targetscan.org) was used to analyze the binding site between miR-34a and PAI-1. Dual-luciferase reporter assay was performed to verify the targeting relationship between miR-34a and PAI-1. Dual-luciferase reporter assay was conducted to verify the relationship between miR-34a and PAI-1. PAI-1 dual-luciferase reporter vectors with or without a mutant miR-34a binding site were constructed and named as PGL3-PAI-1 mut and PGL3-PAI-1 wt, respectively, the Rellina plasmid and the two reporter plasmids were co-transfected into HEK293T cells with the miR-34a mimic plasmid and the NC plasmid, respectively. After 24 h of cell transfection, dual-luciferase reporter assays were performed. Cells in each group were lysed and centrifuged at 12,000 rpm for 1 min to collect the supernatant. The dual-luciferase reporter kit was purchased from Promega and assayed for luciferase activity according to the kit instruction. The 10 μL of lysed cell sample and 100 μL of firefly luciferase working solution were added into the EP tube to measure firefly luciferase activity, and 100 μL of renilla luciferase working solution was added to measure renilla luciferase activity. Relative luciferase activity = firefly luciferase activity/renilla luciferase activity.

### HE staining

The kidney soaked in a neutral formaldehyde solution in each group was collected for making paraffin sections. HE staining was performed after the sections were dewaxed and hydrated. The sections were stained with hematoxylin (Solarbio, Beijing, China) for 2 min followed by washing in tap water. Hydrochloric acid alcohol (1%) was applied for differentiation and rinsed in double distilled (dd) water. Subsequently, sections were stained with eosin (Shanghai Regal Biology Technology Co., Ltd., Shanghai, China) and rinsed in dd water softly before dehydration through gradient alcohol. The sections were then cleared in xylene (Sinopharm Chemical Reagents Co., Ltd., Shanghai, China) and sealed in neutral balsam (Sinopharm Chemical Reagents Co., Ltd., Shanghai, China) followed by being observed under an optical microscope (XP-330, Shanghai Bingyu Optical Instrument Co., Ltd., Shanghai, China) to detect the pathological changes and collagen content in the kidneys. Sections were stained with Masson staining kit (Nanjing Jiancheng Bioengineering Research Institute Co., Ltd., Nanjing, China), and the collagen content of kidney was analyzed according to the instruction of the kit.

### qRT-PCR

The kidneys in each group were collected for making homogenates. Ultra-pure RNA from the homogenate was extracted according to the instruction of Ultra-pure RNA Extraction Kit (D203-01, GenStar Biosolutions Co., Ltd., Beijing, China). RNAs were reversely transcribed into cDNAs according to the instruction of TaqMan MicroRNA Assays Reverse Transcription Primer (4366596, Thermo Scientific, Waltham, MA, USA). Reverse transcription reaction conditions were as follows: reaction at 42 °C for 30–50 min, and 85 °C for 5 s. The primers used to detect miR-34a, U6, PAI-1, and GAPDH were synthesized by Sangon Biotech (Shanghai) Co., Ltd. (Table 1). U6 was taken as the internal reference for miR-34a, and GAPDH as the internal reference for PAI-1. SYBR^®^ PremixExTaq™ II Kit (RR820A, Xingzhi Biotechnology Co., Ltd., Jiangsu, China) was used for quantitative PCR detection. The total reaction system was 50 μL including 25 μL SYBR^®^ Premix Ex Taq™ II (2×), 2 μL forward primer, 2 μL reverse primer, l μL ROX Reference Dye (50×), 4 μL DNA templates, and 16 μL sterilized distilled water. Real-time quantitative PCR was performed using a quantitative PCR instrument (7300, ABI PRISM^®^, Shanghai Kunke Instrument Equipment Co., Ltd., Shanghai, China). Reaction conditions were as follows: pre-denaturation at 95 °C for 10 min, denaturation at 95 °C for 15 s and annealing at 60 °C for 30 s for 40 cycles, followed by extending at 72 °C for 1 min. The 2^−ΔΔCt^ method was used to show the relative expression of the target gene. Each experiment was repeated 3 times. ΔΔCt = (ΔCt_experimental group target gene_ − Ct_experimental group internal reference_) − (ΔCt_control group target gene_ − Ct_control group internal reference_).

**Table 1:** Primer sequence

Gene	Primer sequence (5′–3′)
miR-34a	Forward: GGGTGGCAGTGTCTTAGCT
	Reverse: GTGCAGGGTCCGAGGT
U6	Forward: TCTGTGGAACCCTCCACTCT
	Reverse: GCTTAGGATGCTGCTCCNO
PAI-1	Forward: CAAGCTCTTCCAGACTATGGTG
	Reverse: ACCTTTGGTATGCCTTTCCAC
GAPDH	Forward: TGACCTCAACTANOGGTCTACA
	Reverse: CTTCCNOTCTCGGCCTTG

### Western Blot

Kidney tissue of mice in each group was homogenized. Total protein was extracted using RIPA (R0010, Solarbio, Beijing, China). The transfected cells were washed 3 times with pre-cooled PBS. Appropriate amount of protein lysates (60% RIPA + 39% SDS + 1% protease inhibitor) was added into each cell bottle. The cells were then transferred into EP tubes and lysed on ice for 30 min. Then, the homogenates were centrifuged at 13,500 rpm, 4 °C for 30 min. The supernatants were collected and the protein concentration was measured using BCA kit (Shanghai Ji Ning Industrial Co., Ltd., Shanghai, China). The protein was separated by electrophoresis on a polyacrylamide gel at a voltage of 100 V for 90 min and then transferred to the NC membrane by wet transfer method. The membrane was blocked with 5% BSA at room temperature for 1 h. Then, the membrane was incubated with anti-rabbit PAI-1 (ab66705, 1:1000, Abcam, USA), angiotensin-converting enzyme (ACE, ab254278, 1:1000, Abcam, USA), and ACE2 (ab32137, 1:500, Abcam, USA) overnight at 4 °C, and washed with PBS 5 times for 5 min each time. Subsequently, the membrane was incubated with horse radish peroxidase-labeled goat anti-rabbit IgG antibody (1:5000, Beijing Zhongshan Biotechnology Co., Ltd., China) and developed by the ECL (ECL808-25, Biomiga, USA) at room temperature for 1 min. After the liquid was discarded and plastic wrap was covered, the membrane was photographed with X-ray system (36209ES01, Shanghai Qcbio Science&Technologies Co., Ltd., Shanghai, China). GAPDH (ab9485, 1:251,000, Abcam, Cambridge, MA, UK) was taken as the internal reference. The relative protein expression = the gray value of the targeted protein band/the gray value of the GAPDH protein band.

### ELISA

The serum in each group was taken to detect the content of serum creatinine (Scr), blood urea nitrogen (BUN) and the levels of AngII and Ang1-7. The kidneys (50 mg) in each group were collected for making tissue homogenate and centrifuged to collect the supernatant to detect the content of malondialdehyde (MDA), superoxide dismutase (SOD) and nitric oxide (NO). Optical density (OD) value at 450 nm was measured using a spectrophotometer. The levels of Scr, BUN (Shanghai Xinyu Biotechnology Co., Ltd., Shanghai, China), MDA, SOD, NO (Shanghai Jianglai Biological Technology Co., Ltd., Shanghai, China), AngII and Ang1-7 (Abcam, UK) in each group were measured.

### Statistical analysis

All data were analyzed using SPSS 21.0 software (SPSS, Inc, Chicago, IL, USA). The measurement data were expressed as mean ± standard deviation. The comparison of multiple groups was analyzed by one-way ANOVA, followed by Bonferroni’s post-hoc comparisons. A *P* value less than 0.05 was considered statistically significant.

## Results

### Urinary microalbumin content

**Fig. 1:**
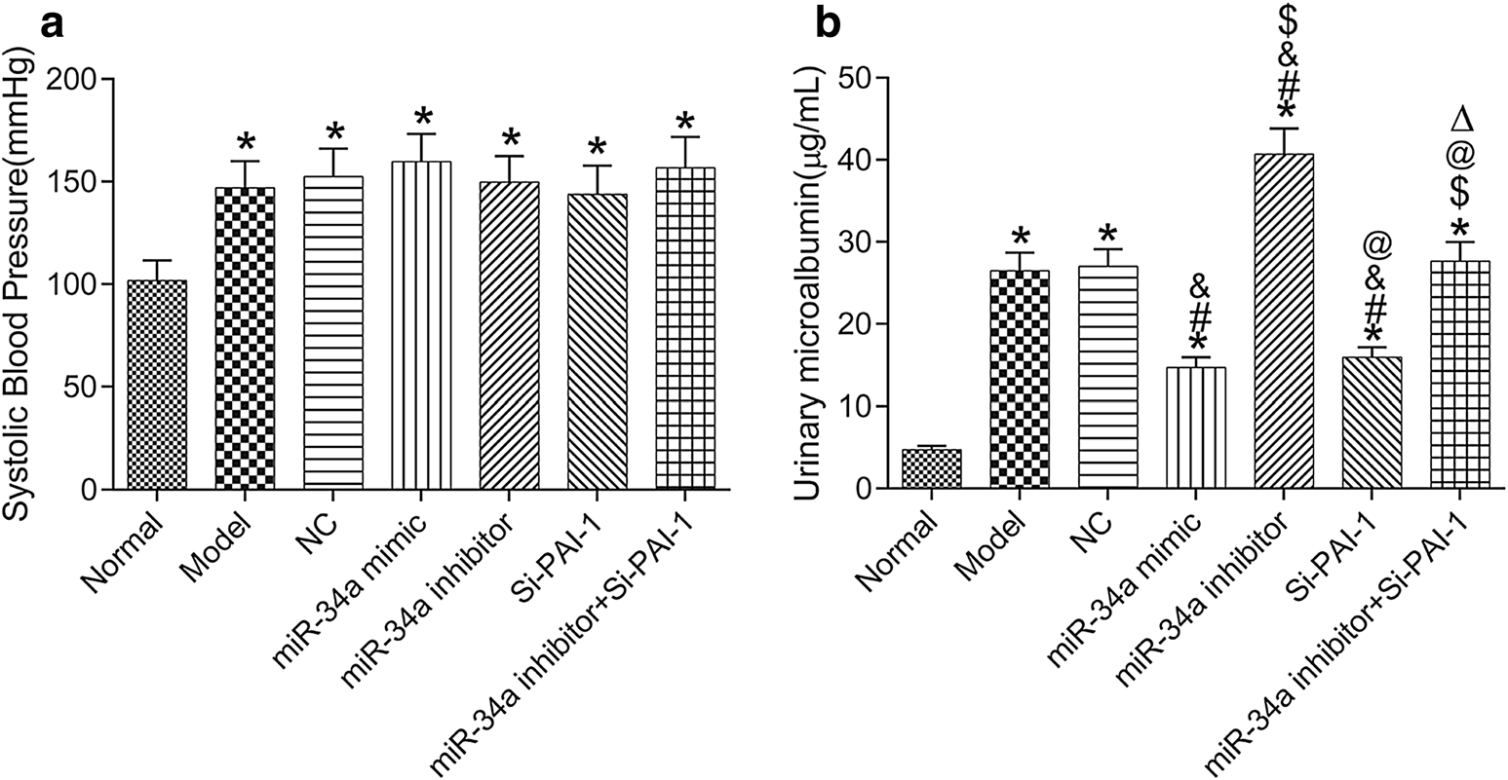
Systolic blood pressure and 24-h urinary microalbumin content in each group. **a** Systolic blood pressure. **b** 24-h urinary microalbumin content. *P < 0.05, compared with normal group; ^#^*P* < 0.05, compared with model group; ^&^*P* < 0.05, compared with NC group; ^$^*P* < 0.05, compared with miR-34a mimic group; ^@^*P* < 0.05, compared with miR-34a inhibitor group; ^△^*P* < 0.05, compared with Si-PAI-1 group. *NC* negative control

Changes in systolic blood pressure in each group were detected (Fig. 1a). Compared with normal group, the systolic blood pressure in the other groups was significantly increased (all *P* < 0.05), and there were no significant differences in systolic blood pressure among the other groups after treatment (all *P* > 0.05). Figure 1b shows the 24-h urinary microalbumin content in each group. Compared with normal group, the 24-h urinary microalbumin was increased in the other groups (all *P *< 0.05) except miR-34a inhibitor + Si-PAI-1 group (*P* > 0.05). Compared with model group, the 24-h urinary microalbumin content was significantly decreased in miR-34a mimic group and Si-PAI-1 group, but significantly increased in miR-34a inhibitor group (all *P *< 0.05). Compared with miR-34a inhibitor group, the 24-h urinary microalbumin content was significantly decreased in miR-34a inhibitor + Si-PAI-1 group (*P *< 0.05).

### Renal histopathology

**Fig. 2:**
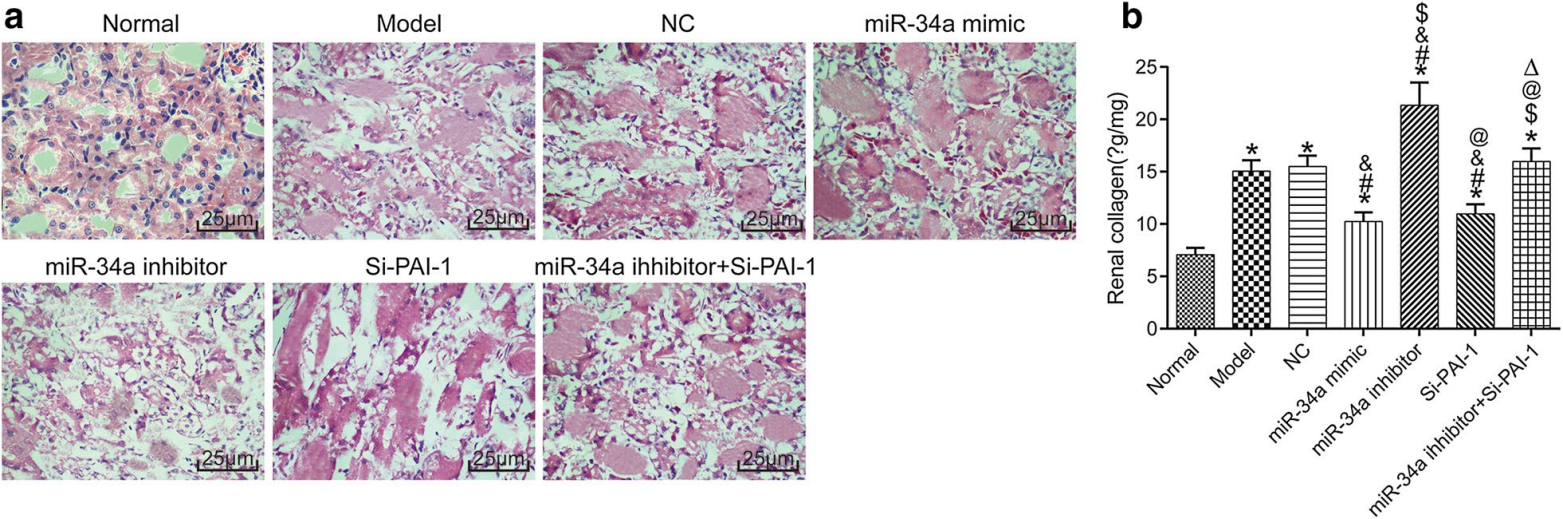
The renal histopathology in each group. **a** HE staining images. **b** Renal collagen content. *P < 0.05, compared with normal group; ^#^*P* < 0.05, compared with model group; ^&^*P* < 0.05, compared with NC group; ^$^*P* < 0.05, compared with miR-34a mimic group; ^@^*P* < 0.05, compared with miR-34a inhibitor group; ^△^*P* < 0.05, compared with Si-PAI-1 group. *NC* negative control

Changes in renal histopathology in each group were detected (Fig. 2a). Groups except normal group showed obvious glomerular structure fibrosis, capillary congestion, focal mesangial hyperplasia, crescentic hyperplasia, and infiltration of inflammatory cells. Figure 2b shows the renal collagen content in each group. Compared with normal group, the renal collagen content was increased in different degrees in the other groups (all *P *< 0.05). Compared with model group, the renal collagen content was significantly decreased in miR-34a mimic group and Si-PAI-1 group, but significantly increased in miR-34a inhibitor group (all *P *< 0.05). Compared with miR-34a inhibitor group, the renal collagen content was significantly decreased in miR-34a inhibitor + Si-PAI-1 group (*P *< 0.05).

### Negative regulation of PAI-1 expression by miR-34a

**Fig. 3:**
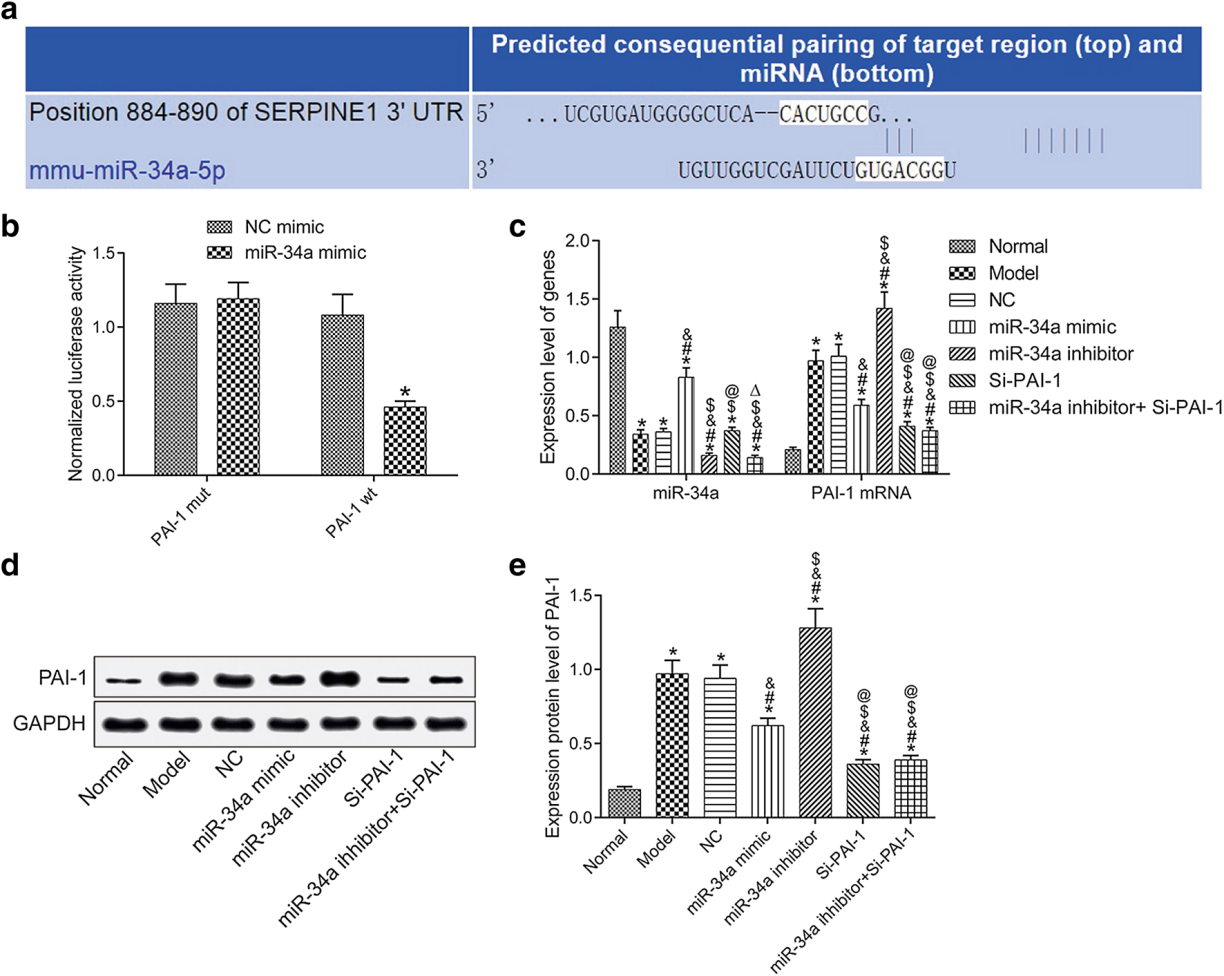
Negative regulation of PAI-1 expression by miR-34a. **a** The binding site between miR-34a and PAI-1. **b** Dual-luciferase reporter assay result. **c** The expression of miR-34a and PAI-1 detection by qRT-PCR. **d** Protein bands. **e** PAI-1 protein expression detection by Western Blot. *P < 0.05, compared with normal group; ^#^*P* < 0.05, compared with model group; ^&^*P* < 0.05, compared with NC group; ^$^*P* < 0.05, compared with miR-34a mimic group; ^@^*P* < 0.05, compared with miR-34a inhibitor group; ^△^*P* < 0.05, compared with Si-PAI-1 group. *NC* negative control

Figure 3a shows the binding site between miR-34a and PAI-1. Figure 3b shows the targeting relationship between miR-34a and PAI-1. Compared with the normal group, the expression of miR-34a in the model group was significantly down-regulated, and the expressions of PAI-1 mRNA and protein were significantly up-regulated (all *P* < 0.05). Compared with the model group, the expression of miR-34a was increased and the expressions of PAI-1 mRNA and protein were decreased in miR-34a mimic group (all *P* < 0.05), but miR-34a inhibitor group showed the opposite results (all *P* < 0.05); there was no significant difference in the expression of miR-34a (*P *> 0.05) but decreased expressions of PAI-1 mRNA and protein (both *P *> 0.05) in Si-PAI-1 group; the expressions of miR-34a, PAI-1 mRNA and protein were decreased in miR-34a inhibitor + Si-PAI-1 group (all *P *< 0.05). See Fig. 3c–e.

### miR-34a inhibits the expression of PAI-1 and improves renal function in hypertensive mice

**Fig. 4:**
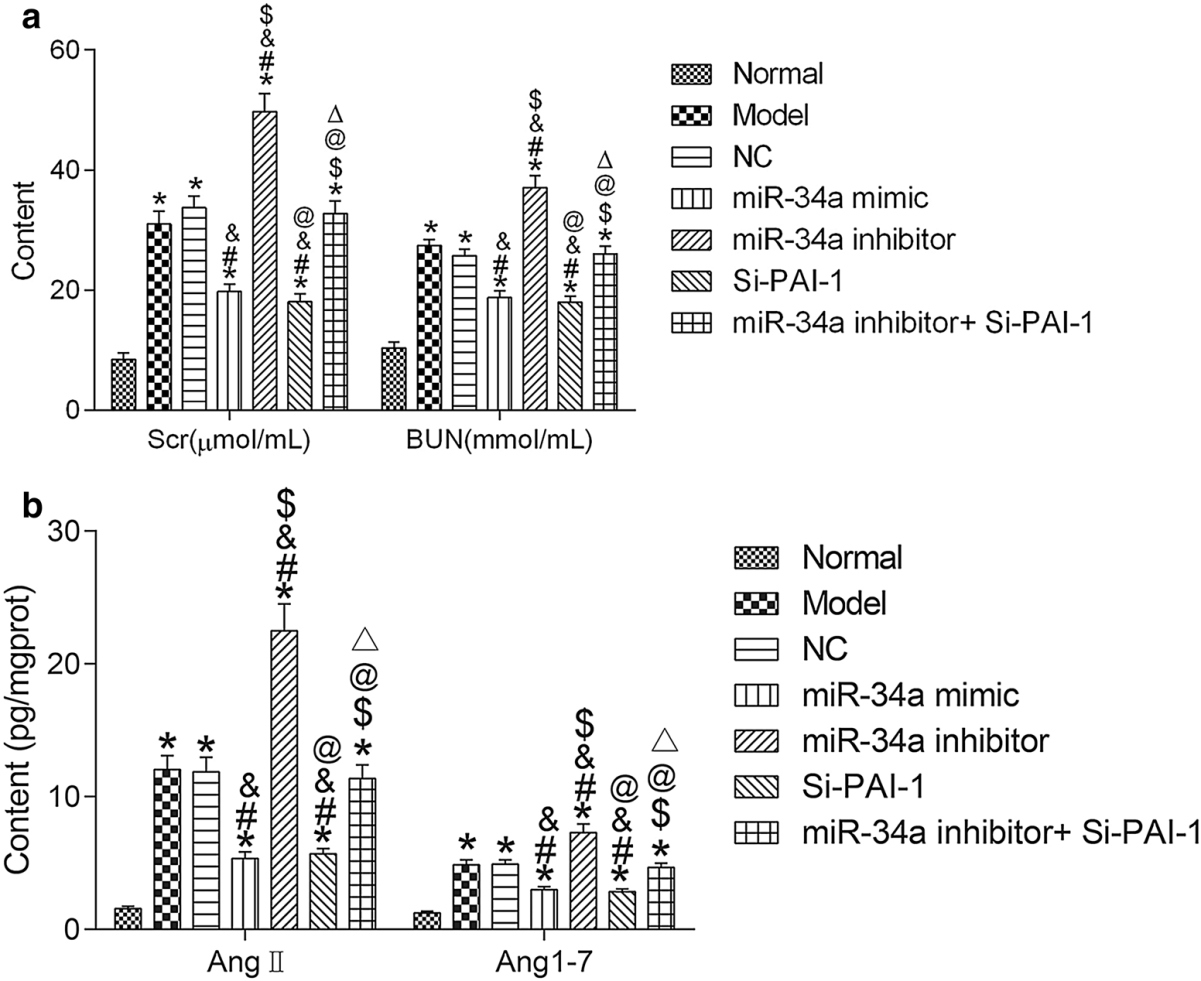
miR-34a improves Scr, BUN, AngII and Ang1-7 by inhibiting the expression of PAI-1. **a** Scr and BUN content. **b** Levels of AngII and Ang1-7 detection by ELISA. *P < 0.05, compared with normal group; ^#^*P* < 0.05, compared with model group; ^&^*P* < 0.05, compared with NC group; ^$^*P* < 0.05, compared with miR-34a mimic group; ^@^*P* < 0.05, compared with miR-34a inhibitor group; ^△^*P* < 0.05, compared with Si-PAI-1 group. *NC* negative control, *Scr* serum creatinine, *BUN* blood urea nitrogen

The content of Scr and BUN of kidney tissue in each group was detected to make out the effects of miR-34a and PAI-1 on renal function in hypertensive mice. The results revealed that the contents of Scr and BUN in hypertensive mice were significantly increased (both *P* < 0.05), those in miR-34a mimic group and Si-PAI-1 group were significantly decreased, and those in miR-34a inhibitor group were significant increased (all *P* < 0.05). Compared with the miR-34a inhibitor group, the content of Scr and BUN in the miR-34a inhibitor + Si-PAI-1 group was significantly decreased (all *P* < 0.05). See Fig. 4a.

Levels of AngII and Ang1-7 were significantly increased in hypertensive mice (both *P* < 0.05) detected by ELISA. Compared with the model group, levels of AngII and Ang1-7 in the miR-34a mimic group and the Si-PAI-1 group were significantly decreased (all *P* < 0.05), while those in the miR-34a inhibitor group were significantly increased (all *P* < 0.05). Compared with the miR-34a inhibitor group, the levels of AngII and Ang1-7 in the miR-34a inhibitor + Si-PAI-1 group were significantly decreased (all *P* < 0.05). See Fig. 4b.

**Fig. 5:**
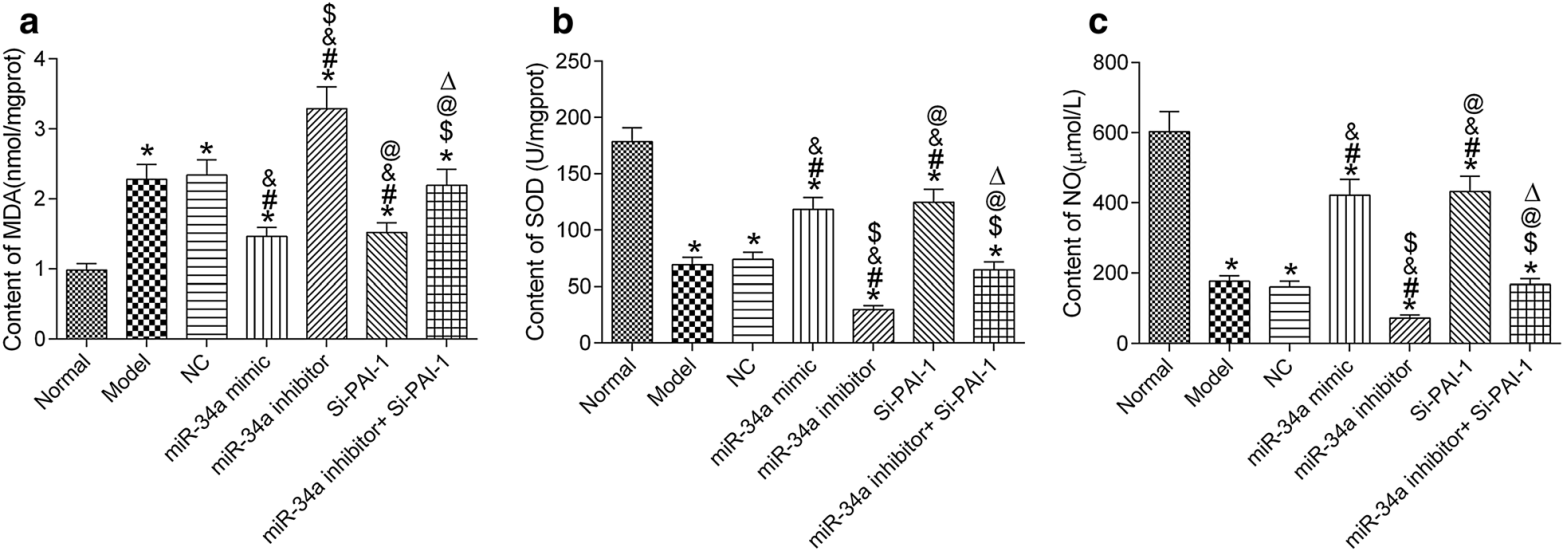
miR-34a improves renal oxidative stress levels by inhibiting the expression of PAI-1 by ELISA detection. **a** MDA content. **b** SOD content. **c** NO content. *P < 0.05, compared with normal group; ^#^*P* < 0.05, compared with model group; ^&^*P* < 0.05, compared with NC group; ^$^*P* < 0.05, compared with miR-34a mimic group; ^@^*P* < 0.05, compared with miR-34a inhibitor group; ^△^*P* < 0.05, compared with Si-PAI-1 group. *NC* negative control, *SOD* superoxide dismutase, *NO* nitric oxide, *MDA* malondialdehyde

The results of MDA, SOD and NO in renal oxidative stress showed that MDA content in hypertensive mice increased significantly, and SOD and NO levels decreased significantly (all *P* < 0.05). Compared with the model group, MDA content in the miR-34a mimic group and Si-PAI-1 group was significantly decreased, but SOD and NO content was significantly increased (all *P* < 0.05); MDA content in the miR-34a inhibitor group was significantly increased, but SOD and NO content was significantly decreased (all *P* < 0.05). Compared with miR-34a inhibitor group, MDA content in miR-34a inhibitor + Si-PAI-1 group was significantly decreased, and SOD and NO content was significantly increased (all *P* < 0.05). See Fig. 5.

**Fig. 6:**
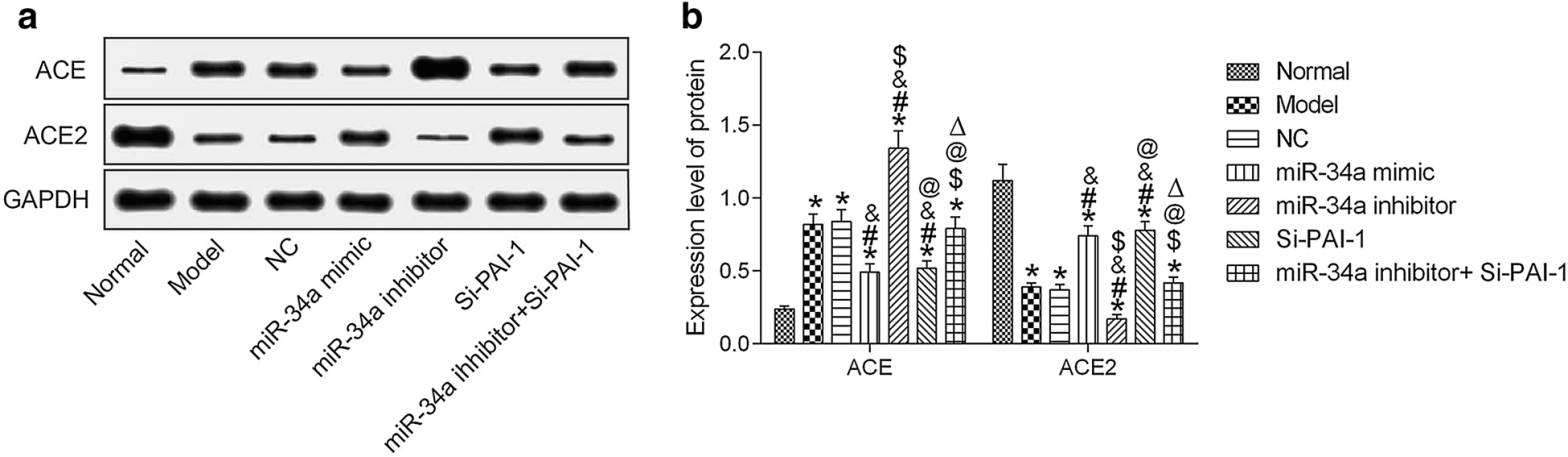
miR-34a improves ACE and ACE2 by inhibiting the expression of PAI-1 by Western Blot. **a** ACE and ACE2 protein expression detection by WB. **b** Quantitative results. *P < 0.05, compared with normal group; ^#^*P* < 0.05, compared with model group; ^&^*P* < 0.05, compared with NC group; ^$^*P* < 0.05, compared with miR-34a mimic group; ^@^*P* < 0.05, compared with miR-34a inhibitor group; ^△^*P* < 0.05, compared with Si-PAI-1 group. *NC* negative control, *ACE* angiotensin-converting enzyme (ACE)

Western Blot was used to detect ACE and ACE2 protein expressions in each group. Compared with the normal group, ACE protein expression was significantly increased in the other groups, while ACE2 protein expression was significantly decreased (all *P* < 0.05). Compared with the model group, ACE protein expression was significantly decreased in the miR-34a mimic group and the Si-PAI-1 group, while ACE2 protein was significantly increased (all *P* < 0.05); the miR-34a inhibitor group showed the opposite results (all *P* < 0.05). Compared with the miR-34a inhibitor group, ACE protein expression was significantly decreased in the miR-34a inhibitor + Si-PAI-1 group, and ACE2 protein was significantly increased (all *P *< 0.05). See Fig. 6.

## Discussion

Due to the social development and changes in people’s lifestyle, the incidence of high blood pressure has been rising each year, and the population with this condition has been growing younger [20]. Hypertension can cause damage to a variety of organs, especially the kidney; renal impairment eventually leads to renal failure, which seriously threatens patients’ lives [21].

In hypertension, renal damage is mainly characterized by glomerular sclerosis which is caused by the accumulation of extracellular matrix [22]. The expression of PAI-1 is significantly elevated in hypertension and promotes the accumulation of extracellular matrix, which in turn aggravates glomerular sclerosis. PAI-1 may mainly cause glomerular sclerosis by affecting angiotensin-converting enzyme [23, 24]. In hypertensive patients, elevated ACE protein expression leads to an increased AngII and Ang1-7 in the kidney. AngII and Ang1-7 promote inflammatory factor synthesis, which aggravates renal stress response and renal damage [25–27]. In hypertensive patients, the level of urinary microalbumin is abnormally elevated. At present, urinary microalbumin content is one of the important indicators for judging whether renal injury occurs in hypertensive patients [28]. A study has shown that ACE2 reduces the urinary microalbumin content after renal damage [29]. In this study, BPN/3J mice were set as the normal group. After Si-PAI-1 injection in some BPH/2J mice, there was no significant difference in the weight loss of hypertensive mice, but the systolic blood pressure and 24-h urinary microalbumin content were significantly reduced. At the same time, we found that ACE protein was significantly decreased in PAI-1 silenced hypertensive rats, while ACE2 protein was significantly increased. Therefore, silencing of PAI-1 may promote the expression of ACE2 and inhibit the expression of ACE, thereby reducing urinary microalbumin content. In addition, the levels of AngII and Ang1-7 were significantly inhibited in hypertensive rats after PAI-1 silencing, and the levels of Scr, BUN and MDA in hypertensive rats after PAI-1 silencing were significantly decreased, but the content of SOD and NO was significantly increased, which was the same as the study of Brown [30]. All results indicated that after PAI-1 silencing, renal damage can be improved in hypertensive mice by regulating ACE, ACE2, AngII and Ang1-7 levels.

Currently, we know very little about the relationship between miR-34a and hypertension. A study has shown that miR-34a is down-regulated in pulmonary hypertension [31]. We found targeted binding sites for miR-34a and PAI-1 via the bioinformatics website, speculating that miR-34a might reduce renal damage in hypertensive mice by targeting inhibition of PAI-1 expression. In this study, after overexpression of miR-34a, blood pressure and 24-h urinary microalbumin content in hypertensive mice decreased; levels of Scr, BUN, MDA, ACE protein expression, AngII and Ang1-7 all decreased; the expression of ACE2 protein, SOD and NO content increased. However, after miR-34a silencing, each indicator showed an opposite result. The effect of silencing of miR-34a was identical to that of microRNA-324-3p [32]. The effect of miR-34a overexpression on hypertensive mice was the same as that caused by PAI-1 silencing. We confirmed that miR-34a can target PAI-1 by performing dual-luciferase reporter assay. In addition, after the combination treatment of miR-34a inhibitor and Si-PAI-1 was applied in hypertensive mice, it was found that PAI-1 silencing can partially improve the blood pressure, urinary microalbumin content and renal damage in hypertensive mice caused by miR-34a silencing.

## Conclusions

In conclusion, the expression of miR-34a is down-regulated in hypertensive mice. The overexpression of miR-34a can targetedly inhibit the expression of PAI-1, which reduces the urinary microalbumin content and improves renal function in hypertensive mice. At present, we only confirm the targeted regulation of miR-34a on PAI-1, and other regulatory networks for miR-34a in hypertension remain unclear. Exploring the regulatory mechanism of miR-34a in hypertension plays an important guiding role in the clinical treatment of hypertension.

## Data Availability

The analyzed datasets generated during the study are available from the corresponding author on reasonable request.
